# General practitioners’ role in improving health care in care homes: a realist review

**DOI:** 10.1093/fampra/cmac071

**Published:** 2022-07-04

**Authors:** Neil H Chadborn, Reena Devi, Claire Goodman, Christopher D Williams, Kate Sartain, Adam L Gordon

**Affiliations:** Unit of Injury, Inflammation and Recovery Sciences, University of Nottingham, Derby, United Kingdom; NIHR Applied Research Collaboration-East Midlands (ARC-EM), Nottingham, United Kingdom; School of Healthcare, University of Leeds, Leeds, United Kingdom; Centre for Research in Public Health and Community Care, University of Hertfordshire, Hatfield, United Kingdom; NIHR Applied Research Collaboration-East of England (ARC-EoE), Cambridge, United Kingdom; Department of Health Sciences, University of Leicester, Leicester, United Kingdom; School of Medicine, University of Nottingham, Nottingham, United Kingdom; Unit of Injury, Inflammation and Recovery Sciences, University of Nottingham, Derby, United Kingdom; NIHR Applied Research Collaboration-East Midlands (ARC-EM), Nottingham, United Kingdom; University Hospitals of Derby and Burton NHS Foundation Trust, Derby, United Kingdom

**Keywords:** end-of-life care, general practice, long-term care facilities, medication review and optimization, nursing homes, quality improvement

## Abstract

**Background:**

Despite recent focus on improving health care in care homes, it is unclear what role general practitioners (GPs) should play. To provide evidence for future practice we set out to explore how GPs have been involved in such improvements.

**Methods:**

Realist review incorporated theory-driven literature searches and stakeholder interviews, supplemented by focussed searches on GP-led medication reviews and end-of-life care. Medline, Embase, CINAHL, PsycInfo, Web of Science, and the Cochrane library were searched. Grey literature was identified through internet searches and professional networks. Studies were included based upon relevance. Data were coded to develop and test contexts, mechanisms, and outcomes for improvements involving GPs.

**Results:**

Evidence was synthesized from 30 articles. Programme theories described: (i) “negotiated working with GPs,” where other professionals led improvement and GPs provided expertise; and (ii) “GP involvement in national/regional improvement programmes.” The expertise of GPs was vital to many improvement programmes, with their medical expertise or role as coordinators of primary care proving pivotal. GPs had limited training in quality improvement (QI) and care home improvement work had to be negotiated in the context of wider primary care commitments.

**Conclusions:**

GPs are central to QI in health care in care homes. Their contributions relate to their specialist expertise and recognition as leaders of primary care but are challenged by available time and resources to develop this role.

**Key messages**

- General practitioners play a critical role in health care in care homes.
- GP’s key contribution is clinical expertise and knowledge of community health care.
- GP’s input into improvement should take account of their competing commitments.

## Background

Around 420,000 people, mostly over 85 years, live in UK care homes. Many have complex long-term health needs related to conditions such as frailty and dementia^1^ requiring input from multiple professionals.^2^ The UK care home sector is shaped by multiple and diverse provider organizations with varying support arrangements from local authorities and the National Health Service (NHS).^3^ Most care homes rely upon general practitioners (GPs) to coordinate residents’ health care. How GPs work with care homes is variable and shaped by local custom and practice, as well as local availability of additional services to supplement or supplant elements of the GP role.^3^ Differences in provision contribute to undesirable variation in health service delivery and outcomes, including rates of unscheduled use of resources.^4^

The NHS England framework for Enhanced Healthcare in Care Homes (EHCH), published in 2016, is a national improvement programme around health care in care homes^5^ due to be fully implemented by 2024. Modifications to EHCH, announced during the COVID-19 pandemic, led to appointment of NHS-employed professionals as care home “clinical leads.”^6^ These roles lacked detailed specification^7^ but were intended to enable engagement between NHS and care home staff, with a focus around health care organization and delivery.

Quality improvement (QI) is defined as activity which drives up quality and is delivered by iterative planning, implementation, measurement, and reflection.^8^ It is widely used in health services. In numerous recent initiatives, GPs have been expected to become involved in QI projects related to care homes because of their role as influential leaders within health care and potentially important partners in improvement.^9^

We used realist review, a theory-driven approach to evidence synthesis^10–12^ to understand what needs to be in place to support GPs in QI in care homes. Realist theories are often expressed as statements that hypothesize how a programme delivers outcomes (O) because of the action of underlying mechanisms (M), that are triggered by particular contexts (C).^13,14^

The present study builds on recent work (The Optimal^13,15^ and Proactive Healthcare in Care Homes [PEACH]^16,17^ studies) which identified key principles of working across health and social care around service development, delivery, and QI.

## Methods

Our realist review sought to explain how the GP role supports (or not) the development and improvement of health care in care homes. Our objectives were to develop a programme theory to explain what is required for GPs to be involved in improvement initiatives in care homes.

The review followed a 4-step approach as outlined in our published protocol.^18^ We made 2 protocol amendments: (i) literature searches focussed on UK studies because of the distinctive features of UK General Practice highlighted in stakeholder interviews; (ii) we used Web of Science instead of ASSIA following information specialist advice. The method and reporting of the study followed the Realist And Meta-narrative Evidence Syntheses: Evolving Standards 2 (RAMESES 2) checklist, shown in Appendix 1. As is common in many realist reviews, we included stakeholder (GP) interviews to explore their understanding of causality when seeking to improve health care in care homes. These findings were used to shape and inform our initial programme theory and hence our literature search terms. This has been described as a way of maintaining theoretical awareness during realist studies,^19^ and establishing putative programme theory in previously published realist reviews.^17,20^

### Step 1: locating possible theories incorporating theory gleaning stakeholder interviews

We used iterative scoping reviews (using broad search terms around care homes and GP) to develop interview guides (Appendix 2). We completed initial semistructured “theory gleaning” interviews (consistent with Abrams et al.^21^) with a purposive sample of GPs drawn from networks developed during previous research. Participants provided written consent. Interviews were semistructured and conducted by telephone or videoconference, recorded using Microsoft Teams software before conversion to MP4 files which were subsequently transcribed by a professional transcription service. Themes for the interview schedule (see Appendix) were based on early reading of the literature and included approaches used by GPs working with care homes to achieve health care improvement; the extent to which improvement objectives were influenced by support and involvement of GPs; and the ways in which this operated through engagement with other professionals. Piloting the interview schedule with the first interview indicated that the schedule was appropriate for the study, and data from this interview were included in analysis. Particular interests or experiences of interviewees were explored in more depth, for example working with pharmacists and providing end-of-life care. Data were organized using data analysis software (NVivo 12; QSR International, Warrington, United Kingdom). Analysis was carried out through iterative process of abduction and retroduction and also referring back to scoping literature. Discussions with the study team (all authors) created putative programme theories, formulated as “if…then…” statements.

### Step 2: searching for evidence

A primary search using terms “*GP and care homes*” (limited to United Kingdom, 2000–April 2020) was conducted across 6 databases (Medline, Embase, CINAHL, Web of Science, PsycInfo, and Cochrane library). We chose this date range because the bulk of the care home literature has been published since 2000,^22^ and because substantial changes in the way that general practice services are designed and delivered to care homes over the last 2 decades^23^ mean that earlier literature would be unlikely to have much relevance to current modes of practice. Two exemplar clinical topics were identified from the GP interviews conducted in step 1 as foci for QI in care homes where GP involvement was particularly important: medicines optimization and end-of-life care. We therefore structured secondary searches for “Medication review” or “optimization and care homes” (limited to United Kingdom, 2010–April 2020) and “end-of-life care” or “palliative care and care homes” (limited to United Kingdom, 2010–April 2020) (see Appendix 3). Citation searches of key authors and included articles were also undertaken. As improvement and development work in care homes is frequently discussed outside academic literature, we also searched grey literature using web searches (Google) looking for outputs from Royal Colleges, specialist societies, and other professional bodies. We made requests for further source material via the Health Foundation “Q Community” and British Geriatrics Society’s Community Geriatrics and General Practice Special Interest Groups.

### Step 3: extracting and organizing data

Articles were included if they described a new service or service model or improvements, with GP involvement. Articles were excluded if they: described routine health care provision outside the context of service development, implementation, or improvement; described social care without health care input; did not describe GPs involvement. Initial screening was conducted by 1 reviewer (NHC) and data were extracted by 2 reviewers (NHC and RD). Remaining team members (CG, CDW, KS, and ALG) reviewed the list of included/excluded articles, the text of included articles, and how these were used to populate the data extraction tool.

Organization and synthesis of data were undertaken using NVivo 12. Full text of included articles as well as grey literature and interview transcripts were imported and coded. Initial codes were based on putative programme theories from step 1, which were modified in response to the evidence. A context expert group was recruited through professional networks and met twice during this step, comprising GP (*n* = 6), care home manager (*n* = 1), pharmacist (*n* = 1), and a care home nurse (*n* = 1). The group discussed emerging programme theories, commented on the study team’s interpretation of evidence and whether “if then” statements and linked CMO configurations (CMOCs) of what was needed for effective GP engagement resonated with their experience. Members were asked to highlight additional relevant evidence for review.

### Step 4: synthesizing evidence and drawing conclusions

In developing programme theories and their constituent CMOCs, we revisited our collated literature to look for evidence that supported or refuted our theories or required further refinement. We then looked for areas of commonality to develop an overall programme theory which encapsulated the range of insights from steps 1–3.

## Results

### Step 1: theory gleaning

Three clinical academic GPs, 3 practicing GPs, and 1 GP commissioner all of whom had worked in and with care homes took part in interviews lasting 28–50 min. Three came from the Midlands, 3 from London, and 1 from the South West offering a range of experiences of working across health and social care in England. The following gives an overview of the practices and infrastructures for general practice in care homes.

Interviews described a range of system prompts, and variations of arrangements at local level, that influenced how GPs interacted with care homes. These were national (e.g. via enhanced services in the General Medical Services contract), and local (e.g. via Clinical Commissioning Groups and Primary Care Networks). Additional funding or alternative contractual arrangements initiated new, possibly short term, ways of working but could also support established working patterns. One example was the move to align practices with care homes, such that all residents within a care home are registered with 1 practice. Formal contracts were felt to give GPs significant latitude in how they prioritized care homes including engaging with QI. Contractual arrangements could support increased frequency of contact from the GP leading to improved relationships with care homes and a shared view of priority topics. This was reinforced if there were regulatory incentives for care homes to participate. Participants noted that the regulator (Care Quality Commission) did not currently seek evidence around QI or partnership working with GPs.

There were a range of views about the extent to which GPs currently participate in or contribute to improvement in care homes. While 1 participant questioned whether GPs, generally, had sufficient expertise in QI, another suggested that GPs were well placed to coordinate improvement projects due to their professional networks. Similarly, there were contrasting views as to whether special interests and expertise in gerontology should be supported or whether GP should focus on generalist practice. Most participants valued multidisciplinary working to improve quality in care homes.

Two clinical areas were exemplar topics of how GP working with care homes could improve resident outcomes. These clinical areas were also identified within scoping searches of the literature: medicines management and end-of-life care. Reflecting on interview transcripts and scoping searches, led to development of putative programme theories, formulated as “if…then…” statements (see Box 1).

**Box 1 Clinical areas identified in step 1 of the review**

**Medicines management**

Participants described projects where pharmacists conducted medication reviews in care homes. Although led by pharmacists, active GP involvement was needed for success and sustainability both to complement shortfalls in pharmacist knowledge and skills (such as assessment of mental capacity) and prescriber acceptance. Trusting relationships amongst the GP, pharmacist, care home staff, and manager were seen as necessary to trigger engagement.

**End-of-life care**

Two participants had been involved in implementing an electronic palliative care system to improve continuity of care for residents approaching the end-of-life. Successful change required trusting relationships and technology for sharing information effectively. This was more likely to be successful when the GP and care home staff viewed this as an appropriate focus.

… if you can get into a good system of making advance care planning routine on admission [to the care home] … the conversation is had, wishes and preferences are gathered. That information is then stored and shared, so obviously we are lucky that we are able to do that. (Participant 21)

### Step 2: searching for evidence

**Fig. 1.**
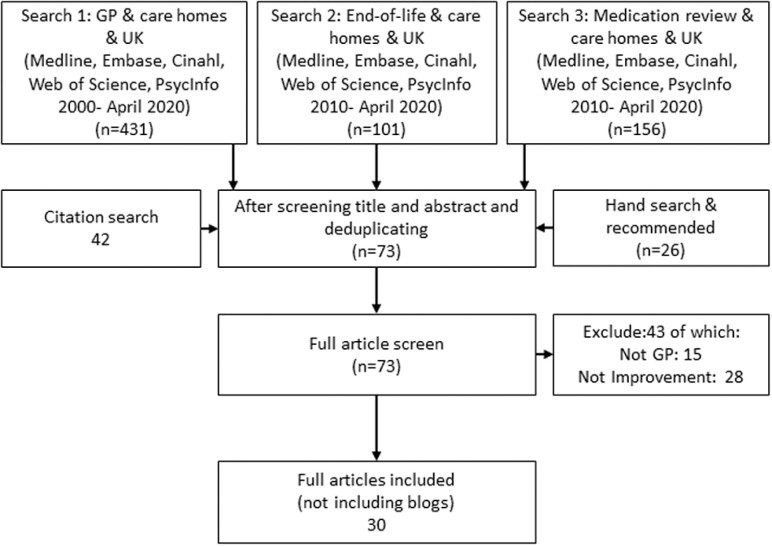
PRISMA-style diagram.

A PRISMA diagram for step 2 is shown in Fig. 1 to capture the primary and secondary searches. After title and abstract screening, 73 articles went forward to full text screening of which 30 were selected for synthesis.

### Step 3: extracting and organizing data

From iterative searches 30 articles and 3 blogs were selected. How we used these data to generate our programme theories is illustrated in detail through an online Appendix 3. Through developing Context-Mechanism-Outcome configurations (CMOCs) for these initiatives we found that 2 broader programme theories emerged, the first was at the microlevel of negotiations and building relationships between practitioners. The second was at the macrolevel of developing the role of the GP and improving how care homes learned to communicate with GPs playing this more consistent role. CMOCs and studies which illustrate them are listed in Table 1.

**Table 1.** Evidence used to establish programme theories.

Programme theory	Context-Mechanism-Outcome configurations	Mechanism
*PT1*: negotiated working with GPs on local improvement initiatives	1. Pharmacist-led medication review: Clinical Medication Review^24^ Care Home Independent Prescribing Pharmacist Study^25^ Shine^26^	Collaborative working with pharmacist
	2. Deprescribing, working with care home staff: Wellbeing Health for People with Dementia^27^	Improved knowledge of deprescribing and collaboration with care home staff
	3. Collaborating for end-of-life care: Evidence-Based Interventions in Dementia—End-of-Life^28^ Audit and review of End-of-Life^29^	Shared understanding with care home staff of sources of uncertainty
*PT2*: developing GP role in regional improvement programmes	4. GP-led end-of-life care: Gold Standards Framework-Care Homes^30^ Difficult conversations^31^	Clarification of role of GP in end-of-life care

### Negotiated working with GPs around local improvement initiatives

We identified QI initiatives which were instigated by other professions, where GPs were invited or requested to support. These involved local negotiations with GPs rather than consideration of the role of the GP and implications for GPs. In these projects, it appeared that GP support was instrumental to the project. However, GP support tended to be assumed with any tensions which then arose, being negotiated post hoc. Where successful resolution was not possible, the GP’s competing responsibilities and willingness to prioritize the work emerged as important limitations.

#### CMOC1—pharmacist-led medication reviews

Medication review projects were the focus of 3 programmes: Clinical Medication Review,^24^ Care Home Independent Prescribing Study (CHIPPS),^25,32^ and Clinico-ethnical framework for multidisciplinary review of medication (funded by Health Foundation, Shine programme). In these initiatives pharmacist independent prescribers supplemented the GP role by collating information from patient records and making prescribing recommendations which were discussed and implemented by the GP.

A key context is the complexity of prescribing for care home residents, particularly those living with multiple long-term conditions. National clinical guidance^33^ and policy framework for Primary Care Networks,^34^ place specific demands on the GP to complete regular medication review in care homes:

…there is little support for practitioners who wish to stop medicines. Solutions to support deprescribing include tools which identify potentially inappropriate medication, such as the STOPP-START tool and Beers criteria.^26^

The CHIPPS study recruited and retained 44 practices to 1 such project.^25^ Success was attributed to selecting GP practices with existing relationships with prescribing pharmacists. This indicates that a trusting relationship enables the mechanism which is that GPs have confidence in the suggestions of the pharmacist. This addresses the challenge of deprescribing; a collaborative practice between GP and pharmacist, leading to deprescribing and improvements such as reduced falls and reduced hospitalizations.

##### CMOC1a

Where a pharmacist has become established and developed a trusting relationship with GP and there is sharing of clinical data (pharmacist can access GP notes and care home care record) (contexts), GP feels confident in the changes proposed by the pharmacist’s medication review (mechanism); GP implements changes in prescriptions including deprescribing (outcomes).

In the “Shine” project,^26^ the team worked flexibly to take account of GP work routines. Four different variations of multidisciplinary communications were arranged to suit the routines of different GPs. Reviews of 38% of residents were discussed with the GP present in the multidisciplinary meeting.^35,36^ A key role for GPs was facilitating shared decision-making with residents and family.^35,36^ We identified that negotiations in arrangements of working patterns led to the mechanism of closer working between GP, pharmacist and residents family in delivering person-centred deprescribing and this could lead to the greatest number of changes to prescriptions (CMOC1b). The “Shine” project led to reduction in polypharmacy with associated cost savings, reduced falls, and reduced hospitalization.^36^

##### CMOC1b

Where a medication optimization project offered a variety of arrangements to match working pattern of the GP practice, this enabled bringing together of pharmacist’s structured medication review and GP’s insights into past medical history and preferences of individual and family (context) leading to both GP and pharmacist contributing to decisions (mechanism) to generate personally tailored and detailed review of medications for residents.

In fact, the evaluation found that cost savings were greatest when GPs did not attend MDT meetings, due to saved GP time, and in this model, GPs were consulted after the MDT. However, the model where GPs attended MDTs led to more changes to medications, which may indicate a more considered approach.^36^

The implication of 1 study was that limited trust between GP and pharmacist was a constraint on the outcomes, because the pharmacist was not able to implement changes independently:

The low implementation rate might have been higher if the pharmacist had been allowed to implement agreed changes^24^

This insight provides a neutral outcome when the context was not suitable to “fire” the mechanism. Fewer medication changes were made compared with the situation where there was a trusting relationship.

#### CMOC2—psychotropic deprescribing led by psychiatrists and clinical psychologists

A second example of a medicine management project is the Well-being and Health for people with Dementia (WHELD) project,^27^ which aimed to deprescribe antipsychotic drugs for residents living with dementia. Prescribing antipsychotics for behavioural symptoms secondary to dementia is associated with adverse outcomes.^37^ The WHELD project centred on training for care home staff to provide social interaction and exercise. GPs were offered training to encourage them to deprescribe antipsychotics, alongside the social intervention:

Physicians were invited to an interactive seminar and/or practice meeting, provided with a toolkit or best practice guide, and given an opportunity for detailed discussion, including scenarios with individual patients.^38^

We have interpreted 2 mechanisms from this set of articles from the WHELD programme. The training, together with the relationship with the care home staff, enabled GPs to feel confident that deprescribing could take place safely, thus GPs could work proactively to deprescribe antipsychotics.

##### CMOC2a

Training GPs and providing information about deprescribing antipsychotics (context) lead to GPs having confidence in deprescribing antipsychotics (mechanism) and hence a reduction in residents being prescribed antipsychotics.

Secondly, staff would be attentive and proactive in their response to residents’ behaviours and were trained in nonpharmacological strategies. Thus, partnership working between GP and care home staff enabled an avoidance of prescription of antipsychotics.

##### CMOC2b

Where training and resources are available for care home staff and staff had built a shared understanding with GPs (context), GPs gain confidence in therapeutic effect of social interventions (mechanism), and signs and symptoms which may have previously triggered GPs to prescribe antipsychotics do not trigger prescription now, due to GPs recognizing that social intervention can be more appropriate than antipsychotics (outcome).

#### CMOC3—GP integral within change initiative to improve end-of-life care

In the third CMOC the focus was on the process of consultation between care home staff and GPs in planning improvements. This configuration was observed in 2 studies, Evidence-Based Interventions in Dementia—End-of-Life (EvIDEM-EoL)^28^ and Audit and Review of Emergency Admissions.^29^ EvIDEM-EoL used appreciative inquiry to explore the uncertainty within teams when decisions had to be made about end-of-life care.^28^ Workshops brought together the care home manager, GP, and district nurse and reviewed findings about end-of-life care in the care home and issues that focussed on who took the lead in care decisions, communication processes, and care routines. In the context of exploring these uncertainties and different practices, the mechanism was that GPs became engaged with planning improvements and committed to change current practice, for example how requests for out-of-hours visits were framed by the care homes and responded to by out-of-hours doctors.

##### CMOC3a

When GP and care home staff have a safe or neutral space to meet to discuss different perspectives and attitudes to end-of-life care that avoids blame (context) this facilitates communication and shared appreciation of clinical processes (tools and frameworks) and shared problem solving (mechanisms) leading to improved access to palliative care for residents and support for staff (outcome).

The audit and review study^29^ involved a presentation of audit data at a multidisciplinary study day with care home managers and staff, GPs, out-of-hours staff, and district nurses. The context of multidisciplinary communication facilitated discussions about preferred place of care and death between residents and family, care home staff, and GPs. Hence, the mechanism was identified as collaborative support between GP and care home staff, with the outcome being a shared approach to care which led to changes in GP attendance at care homes and a reduction in deaths in hospital, indicating an improvement of end-of-life care in the care homes.^29^

The 10% rise in visits by GPs to nursing homes in 08/9 reflects a greater workload which on discussion with the partners is seen to reflect a more active role in anticipatory planning and end of life care^29^

##### CMOC3b

When GPs, care home staff and staff providing out-of-hours care, have the opportunity to discuss together findings of an audit of conveyance to hospital during end-of-life care (context), this encourages a team approach to find ways to improve their coordination of anticipatory care planning (mechanism) leading to an increase in GP visits to nursing homes and a reduction in hospital admissions.

### Securing engagement of GPs in national improvement programmes

We identified 2 examples of nationally coordinated improvement initiatives, focussed on end-of-life care. Firstly, a series of studies described development and evaluation of the “Gold Standards Framework for Care Homes” (GSF-CH) as a structured way to identify people approaching end-of-life and deliver appropriate care.^30^ GSF-CH includes training for care home staff and accreditation of care homes. An important context was the complementarity between the framework deployed in care homes, and the broader GSF programme recently deployed in general practice and incorporated into national monitoring schemes (Quality Outcomes Framework). Therefore, GPs delivery of end-of-life care in care homes was more consistent with their other practice and there was a common understanding with care home staff. There was evidence that care homes accredited with GSF-CH experienced more respectful communication with GPs and hence improved coordination.^39^

#### CMOC4a

Where GPs had recently implemented a new model of end-of-life care (GSF), providing care homes with training and resources consistent with this model (GSF-CH) (context) enabled structured communication between GP and care home staff within this framework (mechanism), which improved collaborative working (outcome).

Secondly, 1 short report described the development and implementation of a programme called “Difficult Conversations.”^31^ Training for GPs in delivering end-of-life care had been codesigned and codelivered by GPs which enabled consistency between the programme and GPs role within care homes. In this training programme, GPs played the role of team members, while the leader was played by the training facilitator. This may provide opportunity for the GP to learn to be the contributor to the team, rather than feeling an expectation to always lead the team.

#### CMOC4b

Where GPs were offered specific training which had been codesigned with GPs, as part of multidisciplinary training programme (context), GPs become more confident in their contribution to the care team (mechanism) leading to improved multidisciplinary working during end-of-life care (outcome).

### Step 4: synthesizing evidence

From the above configurations of CMOCs we further interpreted a programme theory. CMOCs 1, 2, and 3 involve negotiation and trusted relationships between GP and other practitioners (pharmacist and care home staff). This could be described as a microlevel programme theory (PT1) and relies on buy-in by GP, and flexibility of normal roles of the GP to accommodate negotiated working. On the other hand, for CMOC4a, the training interventions may endorse the new role for the GP established by the Gold Standards Framework. Further, the “Difficult Conversations” training (CMOC4b) may have encouraged a new role for GP as contributors rather than leaders of the team. Because this relies on a new model of care for GPs, we describe this as a macrolevel programme theory (PT2).

Despite the recognized importance of GP engagement, few studies described specific measures at design stage to encourage GP acceptance (with the exception of the appreciative inquiry project^28^). Whilst buy-in or “ongoing acceptance” was needed, this was achieved in many cases without planned negotiation with the GP (and practice). There was a sense that GP support was often assumed, indeed in some studies^24,28^ GPs were portrayed as obstructive if they did not engage as envisaged by those leading QI. There was marked variation in the roles that might be required of the GP in different QI projects. Broadly, these related to their role as contract holders, practitioners, prescribers, and/or coordinators of care. Despite their potential value as leaders in QI activity, they were rarely reported to take this role.

## Discussion

GP involvement in improvement initiatives in care homes can lead to improved staff outcomes and systems of working when key contexts are in place that trigger among GPs the following: confidence in care home staff and other health care professionals, a commitment to collaboration, and a fit with existing patterns of working in primary care. GPs were responsible for medical care and access to specialist services. They played a pivotal role in all of the improvement initiatives described, but rarely played a leading or coordinating role. In fact, many of the interventions included in this review actively discouraged this, creating safe spaces where everyone’s views, contributions, and skills were equally valued. The impetus for change invariably came from other professional groups (e.g. pharmacists, palliative care specialists) and GP involvement was always negotiated. The paradox was they were a continuous presence but their contribution as primary care doctors, with unique skills and competencies as part of an MDT, were seldom recognized as key to delivering improvement goals. QI required training to orientate GPs to technical components of an intervention, for example around interventions for behavioural symptoms in people living with dementia, or around communication tools that could span boundaries between care homes and primary care. Building trust and recognizing the GP’s role in carrying forward an initiative were the mechanisms by which GP support could be enlisted for improvement. This was particularly the case when reassuring GPs about mitigating risks of adverse events due to the initiative, for which GPs might ultimately have to assume responsibility.

These findings add to a growing literature on QI in care homes. Innovation and improvement are not new to care homes and there is a rich tradition in the nursing literature of participatory approaches to improvement, including action research and appreciative inquiry.^40^ Structured approaches to QI are, however, less well established in the sector.^16^ Managers and staff have shown that they can adopt and adapt such approaches to improve outcomes for residents,^41^ but without links to the surrounding systems of care, sustaining improvement is difficult.^42,43^ GPs’ input is part of this and is key to bridging the different worlds of organizations and disciplines. How they coordinate or integrate with care homes, however, has not been discussed or explained in detail.^44^ Nor has it been made explicit the kind of resources and investment GPs need to respond effectively as active partners within QI. Here, we have developed programme theories that help describe this. These, though, are ultimately limited by the small amount of available qualitative data gathered by existing articles and project reports—gathering much more rich data in parallel with service evaluation must be a priority for practitioners and researchers working.

The strengths of the study were that we took a systematic approach to searching the academic and grey literature and followed RAMESES guidelines for realist reviews. We were able to bring a disparate and diverse literature together to inform theory development. We used stakeholder interviews and a context expert group to sense-check emerging programme theories and ensure they reflected real-world experience. Our consultation group helped ensure that we captured relevant publications in the grey literature. Our findings are limited by the paucity of description or discussion in the literature on the particular role of GPs in QI in UK care homes. This meant, that while it was possible to see that GPs had been involved in a particular intervention, it became difficult to tease out the precise detail of their involvement. For example, in CMOC1a, it was clear that pharmacists and GPs had to develop collaborative relationships and that this empowered pharmacists to support and challenge GPs’ prescribing. What was not clear was how responsibilities for prescribing were negotiated between professionals over time. In primary care the GP occupies an unusual role of gatekeeper to medical and specialist care but is reliant on multiple health and social care professionals, who are not accountable to them, to support residents’ health care over time.

Our work could also be criticized for its UK focus. This is because much of the international literature failed the relevance test, by virtue of the unique role played by GPs within the UK health care system. The findings from long-term care sectors in the Netherlands and United States, for example, with more evolved approaches to QI were difficult to incorporate. The decision to focus secondary searches on prescribing and end-of-life care could have excluded literature describing other QI projects, however, our consultation group could not identify further literature.

In conclusion, based upon our findings, involvement of GPs is essential to the success of QI initiatives in care homes that are likely to subtend their role and expertise as the coordinators of primary care to residents. It is important to negotiate from the outset their role in the initiative, and how their unique contribution can complement the work of other disciplines. It should not be assumed however that they can or should lead QI and relying on them to do so may create, from the outset, unrealistic expectations. The EHCH roll-out^5^ calls for closer working of GPs with care homes. The appointment of clinical leads during the COVID-19 pandemic is a further indicator of the policy direction to taking more structured responsibility for health care in care homes. However, it could potentially be a mistake to expect GPs to assume such a leadership role without structural support and nominated groups to work with. We identified a consistent need to fit QI activity around GP patterns of consultation.

## Supplementary Material

cmac071_suppl_Supplementary_Material

## Data Availability

The data underlying this article will be shared on reasonable request to the corresponding author.
