# Supplementary material for: General practitioners’ role in improving health care in care homes: a realist review
Source: Fam Pract. 2022 Jul 4;40(1):119–27. doi: 10.1093/fampra/cmac071 (PMC9909664; doi:10.1093/fampra/cmac071)
Supplement: cmac071_suppl_Supplementary_Material [file cmac071_suppl_supplementary_material.docx]

# Online Appendices

# Appendix 1 – RAMESES checklist

This describes the reporting standards according to Realist And Meta-narrative Evidence Syntheses: Evolving Standards 2 (RAMESES 2).

| Section | |  | Page |
| --- | --- | --- | --- |
| 1 | Title |  | 1 |
| 2 | Abstract |  | 2 |
| Intro | |  |  |
| 3 | Rationale | There is a gap in the literature about how GPs get involved in improvement within care homes. There is variability in practice, therefore we sought to identify and synthesise reports where low quality care was identified and addressed, or where best practice was shared and implemented. | 3 |
| 4 | Objectives | “Our objectives were: to develop a programme theory describing contexts where GPs can improve care in care homes; and to describe causative mechanisms whereby GP involvement with care homes leads to service development and improved quality of care. “ | 3 |
| Methods | |  |  |
| 5 | Changes | Changes from initially planned – Search terms focussed on UK only; Web of Science was searched rather than ASSIA | 3 |
| 6 | Rationale for realist | “The care home sector is shaped by multiple and diverse provider organisations and varying support arrangements from local authorities and the NHS[3]. We chose realist review as a methodology sufficiently context-sensitive to account for this variability.” | 3 |
| 7 | Scoping | “Our starting point was theories developed within two prior studies – Optimal[13,15] and PEACH[9,16] – which identified key principles of working across health and social care around service development, delivery and QI. Reference lists from these studies, alongside initial scoping literature reviews using broad search terms around care homes and general practice, helped us structure theory-gleaning interviews with GP leaders and practitioners” | 3 |
| 8 | Search | “Searches were conducted across six databases (Medline, Embase, CINAHL, Web of Science, PsycInfo, Cochrane library), as follows (a full search string is provided in online appendix 3):   - Primary search: GP and care homes, limited to UK, 2000-April 2020 - Secondary search: Medication review or optimisation and care homes, limited to UK 2010-April 2020 - Secondary search: End-of-life care or palliative care and care homes, limited to UK 2010- April 2020 - Citation searches of key authors and key articles” | 3,4 |
| 9 | Selection & appraisal | Screening for inclusion/exclusion was conducted by one reviewer (NC). Data extraction was done by two reviewers (NC, RD). Remaining team members (CG, CDW, KS, ALG) reviewed the list of included/excluded articles, the text of included articles, and how these were used to populate the data extraction tool. Articles were judged on the basis of relevance. | 4 |
| 10 | Extraction | Initial programme theories informed a bespoke data extraction tool including details about study type, intervention, improvement approach, and numbers of care homes, staff, residents and GPs. | 4 |
| 11 | Analysis & synthesis | In step 4, we looked for outcomes in articles which mapped to our emergent Context-Mechanism-Outcome configurations (CMOCs). These largely related to improved partnership working, or improved co-ordination of care processes. For each CMOC, we visited our collated literature to look for evidence that supported our theory, then for evidence that contradicted or required us to adjust it. We focussed on CMOCs where there was evidence from more than one source, suggesting replication of the way in which Contexts triggered Mechanisms to achieve Outcomes (demi-regularities). | 4 |
| Results | |  |  |
| 12 | Flow diagram | PRISMA-style flow diagram.  Iterative searches focused on UK literature and searched for terms related to end-of-life care and medication review. Citation search and hand search of relevant journals was also performed. Searches were carried out in 5 academic databases. | Fig 1; Page 6 |
| 13 | Doc characteristics | Selected documents are listed and range from randomised controlled trials to evaluations of quality improvement initiatives. | Appendix 4 |
| 14 | Main findings | Step 4. We developed two overarching theories. Programme theory 1 was about the embedded relationship and benefits of multidisciplinarity.  Programme theory 2 was about GP leadership at regional or national level. | Table 1, Pages 6-11 |
| Discussion and Conclusion | | |  |
| 15 | Summary | “The main findings of this realist review were that GP involvement in improvement initiatives in care homes led to improved staff outcomes and systems of working…..This was particularly the case when reassuring GPs about mitigating risk of adverse events for which they were nervous about assuming responsibility” | Page 11 |
| 16 | Strengths & weaknesses | “The strengths of the study were that we took a systematic approach to searching the academic and grey literature and followed RAMESES guidelines for realist reviews.…It is possible that our decision to look for case studies in prescribing, end-of-life care and extended role general practice, could have excluded important segments of the literature that could have further developed our theoretical understanding. We did, however, consult regularly with our expert consultee group, who were unable to direct us to other sources.” | 11,12 |
| 17 | Comparison | “These findings add to a growing literature on QI in care homes. …Here we have developed programme theories that help describe this. Each of the initiatives described – around polypharmacy, antipsychotic deprescribing and end-of-life care – could only have achieved their outcomes through GP involvement.” | Pages 12 |
| 18 | Conclusion | “In conclusion, based upon our findings, we recommend that involvement of GPs is essential to the success of QI initiatives in care homes…. relying on them to do so may represent a barrier to success.” | Page 12 |
| 19 | Funding | NIHR HS&DR as stated in Acknowledgement | Page 12 |

# Appendix 2: Interview schedule for theory-gleaning interviews

1. Describe the variety of GP & care home structures
   1. Can you describe the GP-care home ‘link’? ie the working relationship and communication between GP and care home (manager)?
   2. Is it helpful to characterise or categorise different care homes or different types of partnership (GP – care home)?
2. How do GPs work with teams outside of their jurisdiction? For example, out-of-hours care?
   1. Is it similar to the working relationship and communication with the care home, but this time with another team delivering care to the GP’s patients?
3. How do GPs support ‘holistic care’ where aspects may be outside of medical remit of GP?
   1. Within person-centred care or multidisciplinary care are there aspects which the GP may leave to other practitioners or providers (for example social or occupational care), or does the GP take an interest in the oversight of all aspects of care?
4. Please describe medical teaching or training?
   1. How are trainee GPs supported in learning how to deliver high quality care for care home residents?
   2. Are there particular aspects where additional training is available or recommended for GPs?
5. Please describe cross-cutting resources and capacity
   1. Is lack of resource a constraint in the above topics?
6. What outcomes are expected or assessed?
   1. Wellbeing, quality of life (QoL)?
   2. Service use such as hospital admissions?
7. What Information Technology systems are used
   1. Are these integral to projects or additional ‘extras’?
8. Is there anything that I’ve missed, or do you have any additional comments?

# Appendix 3 – Search strategy

Primary search

Search string for Medline

Database: Ovid MEDLINE(R) and Epub Ahead of Print, In-Process & Other Non-Indexed Citations and Daily <1946 to October 18, 2019> (updated to include up to Apr 2020)

Search Strategy:

1 Nursing home/ (8490)

2 nursing home*.tw. (28672)

3 homes for the aged/ (13479)

4 care home*.tw. (3640)

5 or/1-4 [care homes] (47826)

6 Long-Term Care/ (25324)

7 Residential facilities/ (5332)

8 ((long-term or longterm or retir*) adj5 (facility or facilities or institution* or resident*)).tw. (10445)

9 or/6-8 [long term care or residential facility terms] (36831)

10 exp aged/ (3005375)

11 Geriatrics/ (29509)

12 Geriatric Nursing/ (13480)

13 health services for the aged/ (17340)

14 ((older or elder*) adj (person or people or adult* or patient* or inpatient* or resident* or men or women)).tw. (237237)

15 (geriatric* or elderly or seniors or senior citizen* or pensioner* or oldest old or very old).tw. (275140)

16 or/10-15 [old people] (3140511)

17 9 and 16 [long term or residential facilities for older people] (17408)

18 5 or 17 [all care home or long term care or residential facilities for older people] (60893)

19 (general adj (practice* or medical practitioner* or practitioner*)).ti. (33662)

20 (general adj (practice* or medical practitioner* or practitioner*)).ab. /freq=2 (18266)

21 (family adj (medicine or practice* or doctor* or physician* or practitioner*)).ti. (14762)

22 (family adj (medicine or practice* or doctor* or physician* or practitioner*)).ab. /freq=2 (9570)

23 Family Practice/ (64754)

24 physicians, Family/ (16156)

25 Physicians, Primary Care/ (3134)

26 GP.ti. (4505)

27 GP.ab. /freq=2 (22927)

28 (elderly adj2 physician*).ti. (52)

29 (elderly adj2 physician*).ab. /freq=2 (20)

30 Physician Executives/ (4198)

31 medical director*.ti. (706)

32 medical director*.ab. /freq=2 (409)

33 (primary adj1 care doctor*).ti. (195)

34 (primary adj1 care doctor*).ab. /freq=2 (143)

35 or/19-24 [gps or care home doctors] (102790)

36 exp Great Britain/ (357312)

37 (national health service* or nhs*).ti,ab,in. (179827)

38 (english not ((published or publication* or translat* or written or language* or speak* or literature or citation*) adj5 english)).ti,ab. (92722)

39 (gb or "g.b." or britain* or (british* not "british columbia") or uk or "u.k." or united kingdom* or (england* not "new england") or northern ireland* or northern irish* or scotland* or scottish* or ((wales or "south wales") not "new south wales") or welsh*).ti,ab,jw,in. (1973824)

40 (bath or "bath's" or ((birmingham not alabama*) or ("birmingham's" not alabama*) or bradford or "bradford's" or brighton or "brighton's" or bristol or "bristol's" or carlisle* or "carlisle's" or (cambridge not (massachusetts* or boston* or harvard*)) or ("cambridge's" not (massachusetts* or boston* or harvard*)) or (canterbury not zealand*) or ("canterbury's" not zealand*) or chelmsford or "chelmsford's" or chester or "chester's" or chichester or "chichester's" or coventry or "coventry's" or derby or "derby's" or (durham not (carolina* or nc)) or ("durham's" not (carolina* or nc)) or ely or "ely's" or exeter or "exeter's" or gloucester or "gloucester's" or hereford or "hereford's" or hull or "hull's" or lancaster or "lancaster's" or leeds* or leicester or "leicester's" or (lincoln not nebraska*) or ("lincoln's" not nebraska*) or (liverpool not (new south wales* or nsw)) or ("liverpool's" not (new south wales* or nsw)) or ((london not (ontario* or ont or toronto*)) or ("london's" not (ontario* or ont or toronto*)) or manchester or "manchester's" or (newcastle not (new south wales* or nsw)) or ("newcastle's" not (new south wales* or nsw)) or norwich or "norwich's" or nottingham or "nottingham's" or oxford or "oxford's" or peterborough or "peterborough's" or plymouth or "plymouth's" or portsmouth or "portsmouth's" or preston or "preston's" or ripon or "ripon's" or salford or "salford's" or salisbury or "salisbury's" or sheffield or "sheffield's" or southampton or "southampton's" or st albans or stoke or "stoke's" or sunderland or "sunderland's" or truro or "truro's" or wakefield or "wakefield's" or wells or westminster or "westminster's" or winchester or "winchester's" or wolverhampton or "wolverhampton's" or (worcester not (massachusetts* or boston* or harvard*)) or ("worcester's" not (massachusetts* or boston* or harvard*)) or (york not ("new york*" or ny or ontario* or ont or toronto*)) or ("york's" not ("new york*" or ny or ontario* or ont or toronto*))))).ti,ab,in. (1327221)

41 (bangor or "bangor's" or cardiff or "cardiff's" or newport or "newport's" or st asaph or "st asaph's" or st davids or swansea or "swansea's").ti,ab,in. (51805)

42 (aberdeen or "aberdeen's" or dundee or "dundee's" or edinburgh or "edinburgh's" or glasgow or "glasgow's" or inverness or (perth not australia*) or ("perth's" not australia*) or stirling or "stirling's").ti,ab,in. (197789)

43 (armagh or "armagh's" or belfast or "belfast's" or lisburn or "lisburn's" or londonderry or "londonderry's" or derry or "derry's" or newry or "newry's").ti,ab,in. (24417)

44 or/36-43 (2542613)

45 (exp africa/ or exp americas/ or exp antarctic regions/ or exp arctic regions/ or exp asia/ or exp australia/ or exp oceania/) not (exp great britain/ or europe/) (2763442)

46 44 not 45 [UK publications search filter] (2403187)

47 18 and 35 and 46 (201)

Secondary search – Medication review

Database: Ovid MEDLINE(R) <1996 to April Week 3 2020>

1 (medication* adj2 review).tw. (1550)

2 (medication* management or medication* therapy management or medication* strateg*).tw. (3066)

3 "Drug Utilization Review"/ (3465)

4 Medication Therapy Management/ (2056)

5 (drug utili?ation adj2 (review* or evaluat*)).tw. (237)

6 ((multi-drug* or multidrug*) adj2 (therapy or therapies or prescribing or treatment or regime?)).tw. (3142)

7 polypharm*.tw. (5788)

8 polypharmacy/ (4780)

9 (beer* adj1 criter*).tw. (503)

10 ((appropriate or optim* or inappropriat* or suboptim* or sub-optim* or unnecessary or incorrect* or in-correct* or excessive or multiple or concurrent*) adj2 (medicine? or medication* or prescription* or drug*)).tw. (22716)

11 ((over adj1 prescript*) or (overprescrib* or overprescript*)).tw. (1033)

12 ((under adj prescript*) or (underprescrib* or underprescript*)).tw. (326)

13 Inappropriate Prescribing/ (3134)

14 medication appropriateness index.tw. (103)

15 ((prescribing or prescription*) adj2 pattern*).tw. (3478)

16 Drug Prescriptions/ (20426)

17 exp Medication Errors/ (14508)

18 (quality adj (prescribing or prescription* or medication*)).tw. (109)

19 (improv* adj (prescrib* or prescription* or pharmaco*)).tw. (2641)

20 case conferencing.tw. (55)

21 drug regimen review*.tw. (34)

22 pharmacy review.tw. (15)

23 Prescription Drugs/ (5789)

24 pharmacotherap*.tw. (26202)

25 Pharmacists/ (12465)

26 Potentially Inappropriate Medication List/ (432)

27 Medical Overuse/ (1469)

28 or/1-27 [medication review] (112713)

29 Homes for the Aged/ or "home* for the aged".tw. (9412)

30 exp Nursing Homes/ or nursing home*.tw. (30294)

31 "care home*".tw. (2765)

32 Long-Term Care/ (14976)

33 ((long-term or longterm or long-stay or longstay or retir*) adj5 (facilit* or institution* or setting* or resident*)).tw. (12296)

34 Residential Facilities/ (2795)

35 or/29-34 [care homes] (53906)

36 28 and 35 (2338)

37 limit 36 to yr="2010 -Current" (1426)

Secondary search – End-of-life care

Database: Ovid MEDLINE(R) <1996 to April Week 2 2020>

1 end of life.tw. (18018)

2 Terminal Care/ (19827)

3 terminally ill.tw. (3536)

4 Terminally Ill/ (5067)

5 end stage.tw. (50023)

6 dying.tw. (22037)

7 palliat*.tw. (51567)

8 Palliative Care/ (41024)

9 terminal care.tw. (721)

10 Attitude to Death/ (10421)

11 or/1-10 [End of life care] (152986)

12 Homes for the Aged/ or "home* for the aged".tw. (9399)

13 exp Nursing Homes/ or nursing home*.tw. (30257)

14 "care home*".tw. (2759)

15 Long-Term Care/ (14957)

16 ((long-term or longterm or long-stay or longstay or retir*) adj5 (facilit* or institution* or setting* or resident*)).tw. (12271)

17 Residential Facilities/ (2791)

18 or/12-17 [care homes] (53826)

19 11 and 18 (3071)

20 limit 19 to yr="2010 -Current" (1744)

# Appendix 4 – Included articles

| **CMOC** | **Study keyword** | **Date** | **First author surname** | **Title** | **Journal** | **Study type** | **Key findings** | **Ref** |
| --- | --- | --- | --- | --- | --- | --- | --- | --- |
| CMOC1 | Clinico-ethnical framework for multidisciplinary review of medication | 2013 | Baqir | A clinico-ethical framework for multidisciplinary review of medication in nursing homes: A Health Foundation Shine project | International Journal of Pharmacy Practice | Quality improvement | 422 residents were reviewed and 17.4% of medicines were stopped. 6% were stopped due to safety concerns. 2.1% of residents had potential adverse events, but these were reversed. One hour nursing time was released per day due to fewer medications. Care home nurses and GPs were supportive and allowed access to records. | A^1^ |
| CMOC1 | Clinico-ethnical framework for multidisciplinary review of medication | 2013 | Baqir | Blogs | Health Foundation | Quality improvement | Various aspects of quality improvement initiative are described including responding to diversity of GP working practices. | A ^2,3^  35 |
| CMOC1 | Clinico-ethnical framework for multidisciplinary review of medication | 2014 | Northumbria Healthcare NHS FT | Shine 2012 final report. A clinico-ethical framework for multidisciplinary review of medication in nursing homes | Health Foundation | Quality improvement | Various aspects of quality improvement initiative are described including responding to diversity of GP working practices. | 36 |
| CMOC1 | Clinico-ethnical framework for multidisciplinary review of medication | 2014 | Baqir | A clinico-ethical framework for multidisciplinary review of medication in nursing homes | BMJ Open Quality | Quality improvement | Various aspects of quality improvement initiative are described including responding to diversity of GP working practices. | A^4^ |
| CMOC1 | Clinico-ethnical framework for multidisciplinary review of medication | 2017 | Baqir | Impact of medication review, within a shared decision-making framework, on deprescribing in people living in care homes | European Journal of Hospital Pharmacy | Quality improvement | 70.6% of patients had at least one medicine stopped. No significant difference in medicines stopped between pharmacists alone or pharmacist plus GP. | 26 |
| CMOC1 | Medication review | 2006 | Zermansky | Clinical medication review by a pharmacist of elderly people living in care homes--randomised controlled trial | Age & Ageing | Randomised controlled trial | Pharmacist recommended to GP 3.1 drug changes whereas GP alone made 2.4 changes per patient. There was a lower rate of falls in pharmacist-attended residents (0.8) compared to 1.3 falls per patient for the GP group. 75.6% of pharmacist recommendations were accepted, of these 76.6% were implemented. | 24 |
| CMOC1 | Independent prescribers | 2016 | Bond | GP views on the potential role for pharmacist independent prescribers within care homes: Care homes independent pharmacist prescribing study (CHIPPS): 'There has to be something in it for me' | International Journal of Pharmacy Practice | Qualitative interview study | GPs welcomed the pharmacist service. Some concerns about pharmacist initiating medicines. Issues raised: trust, governance and knowledge of older people’s medicine. | 32 |
| CMOC1 | Independent prescribers | 2019 | Inch | The Care Home Independent Prescribing Pharmacist Study (CHIPPS)-a nonrandomised feasibility study of independent pharmacist prescribing in care homes | Pilot and Feasibility Studies | Nonrandomised feasibility study | 44 GP practices and 16 pharmacists were recruited and were retained. 40 residents were recruited and were retained. Outcomes selected were number of falls, drug burden index, hospitalisations, mortality, activities of daily living, and quality of life. The service was well received by care homes and GPs. | 25 |
| CMOC2 | Improving Wellbeing and Health for People  with Dementia (WHELD) | 2016 | Ballard | Impact of WHELD intervention on neuropsychiatric symptoms, antipsychotic use and quality of life in people with dementia living in nursing homes: A cluster randomized trial | American Journal of Psychiatry | Factorial cluster-randomised controlled trial | Reduction of antipsychotic prescription by 50% with a reduced mortality. Social interaction and exercise mitigated the detrimental impact of deprescribing antipsychotics on neuropsychiatric symptoms. | 38 |
| CMOC2 | Improving Wellbeing and Health for People  with Dementia (WHELD) | 2017 | Ballard | Impact of antipsychotic review and non-pharmacological intervention on health-related quality of life in people with dementia living in care homes: WHELD—a factorial cluster randomised controlled trial | Intl J Geriatric Psychiatry | Factorial cluster-randomised controlled trial | Antipsychotic review mainly by GP, as single intervention, led to decrease in health-related quality of life. In combination with social intervention deprescribing antipsychotic did not result in deterioration of health-related quality of life | A^5^ |
| CMOC2 | Improving Wellbeing and Health for People  with Dementia (WHELD) | 2018 | Ballard | Impact of person-centred care training and person-centred activities on quality of life, agitation, and antipsychotic use in people with dementia living in nursing homes: A cluster-randomised controlled trial | PLOS Medicine | Cluster-randomised controlled trial | Improvement in quality of life, agitation and neuropsychiatric symptoms. Rates of prescribing of antipsychotic were low and did not change. | A^6^ |
| CMOC2 | Improving Wellbeing and Health for People  with Dementia (WHELD) | 2020 | Ballard | A Programme of Mixed Methods Research to Develop and Evaluate an Optimized, Fit-For Purpose Person-Centred Intervention to Improve Mental Health and Reduce Antipsychotics amongst People with Dementia in Care Homes (WHELD) | NIHR, Programme Grant for Applied Research | Programme report | Systematic review found four training manuals with RCT evidence. Metasynthesis identified 4 key elements, including antipsychotic review by GP. Factorial RCT showed a reduction in drug use and exercise and social interaction were needed to mitigate detriment of drug withdrawal. Focus groups indicated need for a whole-home approach, including sustained relationships. A 9month RCT showed improvement in quality of life, agitation and neuropsychiatric symptoms. Health and social care costs were reduced. Delivering the intervention required a flexible approach. An e-learning module was developed for GPs. | 27 |
| CMOC3 | Evidence-Based Interventions in Dementia – End-of-Life | 2016 | Amador | Evaluation of an organisational intervention to promote integrated working between health services and care homes in the delivery of end-of-life care for people with dementia: understanding the change process using a social identity approach | International Journal of Integrated Care | Appreciative enquiry | The intervention supported integrated working due to shared goals and recognition of different expertise.  Bottom-up process of implementing context-specific practice innovations and tools | 28 |
| CMOC3 | Audit and Review of Emergency Admissions (end-of-life care) | 2011 | Evans | Factors influencing emergency hospital admissions from nursing and residential homes: positive results from a practice-based audit | Journal of Evaluation in Clinical Practice | Quality improvement - audit | Initial audit showed 55% of deaths occurred in the care home, whereas second audit, this had increased to 75.5%. GP visits to nursing home patients increased 10.3%, but visits to residential home patients decreased 5.4% There was a 43% reduction in emergency admissions with a 45% decrease in deaths in hospital. | 29 |
| CMOC4 | Difficult conversations | 2018 | Brighton | 'Difficult Conversations': evaluation of multiprofessional training | BMJ supportive & palliative care | Evaluation | 655 participants including GP, nurses, social care staff, allied health professionals and care home staff. All groups showed increased self-confidence, knowledge and skills. They appreciated interprofessional learning. | 31 |
| CMOC4 | Gold Standards Framework - Care Homes (GSF-CH) | 2009 | Badger | An evaluation of the implementation of a programme to improve end-of-life care in nursing homes | Palliative Medicine | Evaluation | Significant improvements in end-of-life care, increase in proportion of residents who had an advance care plan and increase in proportion of residents who died in the care home. Crisis admissions to hospital were reduced. | A^7^ |
| CMOC4 | Gold Standards Framework - Care Homes (GSF-CH) | 2011 | Hall | Implementing a quality improvement programme in palliative care in care homes: a qualitative study | BMC Geriatrics | Qualitative study of QI | Benefits: Improved symptom control, team communication, external support (including GP), staff confidence, residents’ choice, reputation of home. Barriers: Increased paperwork, lack of knowledge, costs, problems with lack of cooperation of GP. | 39 |
| CMOC4 | Gold Standards Framework - Care Homes (GSF-CH) | 2012 | Badger | An evaluation of the impact of the Gold Standards Framework on collaboration in end-of-life care in nursing homes. A qualitative and quantitative evaluation | International Journal of Nursing Studies | Qualitative and quantitative evaluation | Challenges to collaboration: working with many GPs, poor access to out-of-hours and specialist services. Improved collaboration was identified by 33% of managers. Staff reported increased knowledge, confidence, discussing care with GP and palliative specialists. | 30 |
| CMOC4 | Gold Standards Framework - Care Homes (GSF-CH) | 2014 | Kinley | The provision of care for residents dying in UK nursing care homes | Age & Ageing | Review of deceased care records | Dependency of residents has increased with 56% dying within a year of admission. Within the last 6 months of life, support from healthcare specialists was variable. | A^8^ |
| Insufficient rigour, relevance or richness | Medication review | 2000 | Khunti | Effect of systematic review of medication by general practitioner on drug consumption among nursing-home residents | Age & Ageing | Evaluation | 51% of patients had a medication stopped and 26% of patients changed to a cheaper alternative or reduced dosage. | A^9^ |
| Insufficient rigour, relevance or richness | Medication review | 2003 | Hughes | Information is care: the need for data to assess the quality of care in UK nursing and residential homes | Expert Opinion on Drug Safety | Expert opinion | There is a lack of data on prescribing. | A^10^ |
| Insufficient rigour, relevance or richness | Fracture prevention | 2008 | Cox | Educating nursing home staff on fracture prevention: a cluster randomised trial | Age & Ageing | Cluster randomised trial | There were no differences between the intervention and control in primary outcomes; incidence of total fractures or total hip fractures, no differences were found for falls or hip protector use. An increase in prescription of bisphosphonate, calcium and vitamin D was observed in the intervention group. | A^11^ |
| Insufficient rigour, relevance or richness | Medication review | 2011 | Patterson | A cluster randomized controlled trial of an adapted U.S. model of pharmaceutical care for nursing home residents in Northern Ireland (Fleetwood Northern Ireland study): a cost-effectiveness analysis | Journal of the American Geriatrics Society | Cluster randomised controlled trial | Psychoactive drugs were taken by fewer residents after 12months of the intervention (19.5%) compared to control (50%). No differences in falls rates were observed. | A^12^ |
| Insufficient rigour, relevance or richness | Quality & Outcomes Framework | 2011 | Shah | Quality of chronic disease care for older people in care homes and the community in a primary care pay for performance system: retrospective study | BMJ | Quantitative retrospective | Quality indicators of chronic disease care were lower for residents of care homes than the community over 14 out of 16 indicators (after adjustment). Residents of care homes were more likely to be excluded by GPs from targets within the Quality and Outcomes Framework. | A^13^ |
| Insufficient rigour, relevance or richness | Nutrition review | 2014 | Madigan | A cluster randomised controlled trial of a nutrition education intervention in the community | Journal of Human Nutrition & Dietetics | Cluster randomised controlled trial | GPs and nurses who attended an educational intervention about supporting patients following discharge from hospital showed greater knowledge than the control, although this was not sustained at 6 months. | A^14^ |
| Insufficient rigour, relevance or richness | Palliative care | 2016 | Iliffe | Improving palliative care in selected settings in England using quality indicators: a realist evaluation | BMC Palliative Care | Realist evaluation | GP could not be recruited. Care homes could not be retained. The quality indicator set were not motivating for GP or care homes; they may have been too specialist. | A^15^ |
| Insufficient rigour, relevance or richness | Antipsychotic deprescribing | 2016 | Szczepura | Antipsychotic prescribing in care homes before and after launch of a national dementia strategy: an observational study in English institutions over a 4-year period | BMJ Open | Quantitative retrospective analysis | Prescribing rates did not change following implementation of the policy, nor was there a shift to second-generation antipsychotics. Duration of prescribing was excessive in 69.7% of cases. Association between high prescribing and deprivation of the area of the care home. Association between low prescribing and single GP practice. | A^16^ |
| Insufficient rigour, relevance or richness | Stroke | 2017 | Sadler | Shaping innovations in long-term care for stroke survivors with multimorbidity through stakeholder engagement | PLoS ONE | Qualitative study | Among 37 participants, including GPs, selected the following purposes as priorities for data improvement: continuity of care, mental health, access to health and social care, multiple risk factors. From this consultation a decision support tool was co-designed. | A^17^ |
| Insufficient rigour, relevance or richness | New care models | 2018 | Elvey | Implementing new care models: learning from the Greater Manchester demonstrator pilot experience | BMC Family Practice | Qualitative observation of pilot projects | Challenges identified were trust to enable collaboration, especially relating to data sharing. | A^18^ |
| Insufficient,  rigour, relevance or richness | CMHT | 2018 | Stewart | Provision and perceived quality of mental health services for older care home residents in England: a national survey | International Journal of Geriatric Psychiatry | Survey | Only 18% of community mental health teams for older people allocated staff for care homes, and services varied. 40% of teams provided training to care homes staff. Service manager was likely to report their service as good if the service had a systematic process for reviewing mental health and antipsychotic prescriptions, including contact with a GP. | A^19^ |
| Insufficient rigour, relevance or richness | Vanguard | 2018 | Stocker | Care home services at the vanguard: a qualitative study exploring stakeholder views on the development and evaluation of novel, integrated approaches to enhancing healthcare in care homes | BMJ Open | Qualitative study of planned implementation | There was a moral imperative for proposed change to services. However integrated working was not clearly understood. There was a perception of the programme being top-down and imposed by the health service. Trust within the system was valued. | A^20^ |

Table 1 Included articles and blogs. Where articles have been cited in the main text, the citation number is consistent, where articles have not been cited, they are denoted with “A superscript” and listed in the reference list below. Abbreviations: EvIDEM-EoL; Evidence-Based Interventions in Dementia – End-of-Life, WHELD; Well-being and Health for people with Dementia, GSF-CH; Gold Standards Framework for Care Homes, CMHT; Community mental health teams

# Appendix 5 Selected excerpts from articles and quotes from interviews used to inform CMO development

| CMO | Description | Excerpt or quote | Reference or Interview |
| --- | --- | --- | --- |
| CMO1 | C: Problem of inappropriate prescribing | Seventy percent of care home residents experience at least one medication error. In addition 50% of medicines are not taken as prescribed, with adverse drug reactions contributing to 17% of all hospital admissions. | A^21^ |
| CMO1 | C: Problem of inappropriate prescribing | …excess medicines (sometimes inappropriate), lack of structured review of medicines, communication issues: many residents were unaware of what treatment they are on, long medication rounds and timing of rounds not resident-centred | 26 |
| CMO1 | C: Problem of inappropriate prescribing | Whilst there are clear guidelines for starting medicines; there is less guidance for stopping medicines. The issues can be summarised as: Prescribers face a number of ethical, legal and professional challenges when considering stopping medicines. Residents are usually not involved in decisions about medicines prescribed to them. | 26 |
| CMO1 | C: Problem of inappropriate prescribing | in six weeks time when I have got the notes, have I got the time then to do the medication review properly. So, they are all potential areas of error. And then they go into hospital, and you know someone comes out of hospital there has been a medication change that hasn’t gone on a discharge summary. So, to me having the care home pharmacist who liaising with the home, liaises with the manager | #21 |
| CMO1 | C: Problem of inappropriate prescribing | general practitioners do not review most care home patients’ medication. | 24 |
| CMO1 | C: Problem of inappropriate prescribing | Our pre-baseline annual review rate was 24%, which is lower than in our previous study in older people living in their own homes (44%). Three-quarters of this vulnerable group are not having their medication reviewed. | 24 |
| CMO1 | C: complexities | decisions are based on having conversations around context and I don’t know many pharmacists who are sighted on being like the Mental Capacity Act and how we should be making decisions based on a person’s wishes and preferences or who is the legal decision maker authority is and then having a conversation | #16 |
| CMO1 | C: complexities | they are so thin on the ground, they haven’t got the capacity to go back into review. So actually, a GP will say, they are asking me to stop that but actually I don’t know why they’re on it, it may cause symptoms, they may be worse off of it, so actually I’m going to leave them on it. But so, unless the pharmacist is working at their highest level of their competency, then either they are unsafe or the workload falls back on primary care anyway. | #16 |
| CMO1 | C: complexities | Deprescribing is difﬁcult with few tools, guidelines and little evidence to support practitioners | 26 |
| CMO1 | C: multidisciplinary work* | the approach they tested involved getting people together to discuss treatment and make decisions. Where possible, the group included the care home resident, a family member, a nurse from the care home, a GP and a pharmacist | 24 |
| CMO1 | C: multidisciplinary work* | Getting the right team together was important. The team included health professionals or managers from GP practices and care homes, experts in psychiatry, Age UK, and the executive management team. | 24 |
| CMO1 | C: multidisciplinary work* | The medication reviews were conducted by clinical pharmacists, with the findings discussed by multidisciplinary teams which included care home nurses and GPs as well as patients, families and carers where this was possible. | A^3^ |
| CMO1 | C: multidisciplinary work* | A pharmacist undertook detailed medication reviews using primary care records and the results were discussed at a multidisciplinary team (MDT) meeting involving the care home nurse and the resident’s general practitioner (GP), with input from the local psychiatry of old age service (POAS) where appropriate. | A^21^ |
| CMO1 | C: multidisciplinary work* | followed by a MDT meeting involving pharmacists and care home nurses, with other professionals (eg, general medical practitioners and mental health professionals) joining when required. At the MDT, the information from the pharmacist-led review was discussed and an action plan was formulated | 26 |
| CMO1 | C: multidisciplinary work* | One of the key concerns practices had was the capacity to release GPs to attend the MDT. However, our differential analysis of the models showed that GP involvement in the MDT resulted in the greatest interventions. | 24 |
| CMO1 | C: multidisciplinary work* | we will set aside an hour to go through … out of the 40 bedded home, that she will do a medication review round maybe twice a year… We then set aside an hour where we will go through the ones where she is considering stopping, or this person has got this illness and they haven’t got this drug. And so, she doesn’t make any of the decisions on her own, those decisions are made with me with my clinical knowledge of the patient. | #21 |
| CMO1 | C: Policy | The National Service Framework for Older People proposed (without citing evidence of its value) regular review of care home residents and their treatment. | 24 |
| CMO1 | Context where mechanism may not fire: Risk of complaints | the risk is that people say well, living with significant frailty, they are over 85, we’re going to stop their statins. Now some people would say that, some people would defend that but then if you haven’t had that conversation with a family member and then they have a stroke the next week because you’ve stopped their statins, then that opens up all sorts of avenues for complaints. | #16 |
| CMO1 | C: Structured process* | We collected clinical data from GP records. The criterion for a medication review having occurred was if the term ‘medication review’ or ‘drug review’ or a similar phrase was recorded. | 24 |
| CMO1 | C: Structured process* | She obtained the number of falls from the homes’ official accident book. The details of medication were from GP records. | 24 |
| CMO1 | C: Structured process* | review of the GP clinical record and a consultation with the patient and carer. The pharmacist formulated recommendations with the patient and carer and passed them on a written proforma to the GP for acceptance and implementation. GP acceptance was signified by ticking a box on the proforma. | 24 |
| CMO1 | M: Trusted relationship | if you’ve got a very good pharmacist, I think GPs are only too happy for them to do some of the work | #16 |
| CMO1 | M: Trusted relationship | clinical pharmacists involved were experienced independent prescribers competent to make autonomous decisions | A^21^ |
| CMO1 | M: Trusted relationship | It became apparent very quickly that, for our proposal to work, we needed care homes, GPs and patients to start working together when making decisions about medicines – the project has really brought these groups together…Better relationships have meant better care for patients in care homes. | A^2^ |
| CMO1 | M: Trusted relationship | that model has the potential to be excellent, provided that those AHP’s are well aligned, trained by, trusted by their teams. I’ve worked with some brilliant AHP’s, but where it works well is when they’re really well embedded into an organisation and they really know each other’s skills and strengths and weaknesses. In particular they know what they don’t know. | #19 |
| CMO1 | M: Trusted relationship | having the care home pharmacist who liaising with the home, liaises with the manager does the first review on a new patient, and then also does a review when someone has been discharged from hospital to reconcile the meds. Who then involves me, so does the research, does the … you know has been … it was my one area that as a committed GP I previously felt I wasn’t doing to the standard I wanted to do it. So, having that extra support, but it is still a team, you know and obviously the pharmacist won’t be there all the time. | #21 |
| CMO1 | M: Trusted relationship | An existing arrangement with a PIP [pharmacist independent prescriber]… was preferred….final selection prioritised practices that had an established working relationship with a PIP. | 25 |
| CMO1 | M: Trusted relationship | We also created better links between experts in old age psychiatry and general practice, so decisions that ordinarily wouldn’t have been made (eg stopping 'specialist' medicines such as antipsychotics in dementia patients) could be openly discussed. Better relationships have meant better care for patients in care homes. | A^2^ |
| CMO1 | M: Tailoring to diverse GP working practices | ‘We were naïve in thinking we could come up with a model and apply it across the whole health economy. Every practice was different,’ Wasim says. To overcome this, the team came up with several different ways of involving GPs, so that GPs could be part of every review. | 35 |
| CMO1 | M: Tailoring to diverse GP working practices | Over several cycles four potential models of working with GPs were developed: 1) GP attended the MDT, joint decisions made with the care home nurse and pharmacist, ,. | A^21^ |
| CMO1 | M: Tailoring to diverse GP working practices | …models of working with GPs … 2) Interventions discussed with the GP after pharmacist review and prior to the MDT, | A^21^ |
| CMO1 | M: Tailoring to diverse GP working practices | …models of working with GPs … 3) Interventions discussed with the GP following the MDT but prior to resident involvement | A^21^ |
| CMO1 | M: Tailoring to diverse GP working practices | … models of working with GPs … 4) No GP involvement, prescribing pharmacist leads the process. Interventions recorded in the general practice electronic notes, GPs could challenge the interventions | A^21^ |
| CMO1 | M: Tailoring to diverse GP working practices | Individual medical practices work differently ... Through consultation with lead GPs and practice managers we identified four models of GP involvement | A^22^ |
| CMO1 | M: Tailoring to diverse GP working practices | some GP practices won’t take on a care home, you know they won’t do it for love nor money. And you will get other GP practices that might look after 6 or 8, and we have got those two extremes within [our locality]. | #21 |
| CMO1 | O: Prescriptions changed | The patient’s GP accepted 75.6% (565/747) of the pharmacist recommendations. Of the accepted recommendations, 76.6% (433/565) were acted upon | A^23^ |
| CMO1 | Lack of outcome – recommendations rejected by GP | The GP did not implement 23.4% (132/565) of the accepted recommendations, however. Over 7% (52/747) recommendations were rejected by the patient’s doctor. The overall implementation rate of recommendations was therefore 58% (433/747). The low implementation rate might have been higher if the pharmacist had been allowed to implement agreed changes. | A^23^ |
| CMO1 | O: Cost saving | The net cost savings are £21,705 or £206 per patient reviewed. | A^2^ |
| CMO1 | O: Reduction in falls | There was a large and significant reduction in the number of falls (0.8 falls per patient in the intervention group, compared with 1.3 in the control group). | A^23^ |
| CMO1 | O: Safety monitoring | nine adverse effects reported … events were discussed with GP colleagues and not deemed serious and they were identiﬁed and appropriately rectiﬁed by either monitoring the patient or reinstating the drug or an alternative…. | 26 |
| CMO1 | O: Safety monitoring | …appropriate monitoring post-cessation is important to pick up any untoward effects that may occur. One of the limitations in attributing adverse effects to the cessation of a drug is that is it difﬁcult to be certain of causation, bearing in mind the complexity of comorbidities in the older population… | 26 |
| CMO1 | O: Safety monitoring | Of the intervention patients, 42% (139/331) required a test to monitor their condition and/or their medicines. For 24% (161/672) of medicine-related interventions, a test was recommended, 13.7% (23/161) of these resulting in a change in medication. | 24 |
| CMO1 | Neutral outcome of number of medicines prescribed | two models of delivery for the service (pharmacist actioning decisions or pharmacist and GP actioning decisions), there was no statistical difference in number of medicines deprescribed between the two methods.. | 26 |
| CMO1 | O: Reduction in hospital admissions | We found a statistical reduction in hospital admissions – people were actually less likely to go into hospital after having one of our interventions | 35 |
| CMO1 | Neutral outcome of hospitalisation | There is no significant change in consultations, hospitalisation, mortality, SMMSE or Barthel scores | 24 |
| CMO2 | C: Evidence-based guidelines | Antipsychotic review focused speciﬁcally on the review of antipsychotic prescriptions by primary care physicians or psychiatry specialists, based on the National Institute for Health and Clinical Excellence dementia guidelines and facilitated by antipsychotics guidance developed by the Alzheimer’s Society in partnership with the U.K. Department of Health. | A^24^ |
| CMO2 | C: Evidence-based guidelines | The guidelines emphasized careful medical assessment of underlying causes of neuropsychiatric symptoms such as pain and factors leading to delirium, the use of monitoring and/or nonpharmacological interventions as a ﬁrst-line approach before considering pharmacotherapy… | A^24^ |
| CMO2 | C: Training for care home staff | Seminars were conducted for care staff regarding safe antipsychotic prescribing, monitoring, and review | A^24^ |
| CMO2 | C: Seminar for GP | Physicians were invited to an interactive seminar and/or practice meeting, provided with a toolkit or best practice guide, and given an opportunity for detailed discussion, including scenarios with individual patients. | A^24^ |
| CMO2 | C: MDT meeting * | Therapists also worked with physicians and staff to augment person-centered care during antipsychotic withdrawal. | A^24^ |
| CMO2 | C: Structured process* | WHELD therapists worked with the champions and other staff to develop processes to prompt physician review according to best practice guidelines. | A^24^ |
| CMO2 | M: GP as decision-maker | Prescribing decisions were still made entirely by the participants’ own physician. In the majority of cases this was the primary care physician. | A^24^ |
| CMO2 | O: Antipsychotic review reduced use | The main factorial study demonstrated that antipsychotic review significantly reduced antipsychotic use by 50% (OR 0.17, 95% CI 0.05 to 0.60). The intervention of antipsychotic review plus social interaction significantly reduced mortality (OR 0.26, 95% CI 0.13 to 0.51) compared with the group receiving neither antipsychotic review nor social interaction. | 27 |
| CMO3 | C: National policy –enabling dignified and peaceful death | [aim of QI] to reduce emergency admissions and enable a digniﬁed and peaceful death in the care home setting, as envisaged in both the National Dementia Strategy [Ref] and the End of Life Care Strategy [Ref]. | 31 |
| CMO3 | C: Continuity diminishing | …when I first started in general practice, I really knew all my patients and their families and there was continuity of care and currently continuity of care is diminishing rapidly and that in itself is a potential real problem. | #19 |
| CMO3 | C: Recognising the dying phase | … when you’re dealing with COPD, heart failure, diabetes, dementia and all of those things and then I think there’s that final, the dying phase, so the last weeks to days, hours of life where it’s then clear that the person isn’t going to recover and is dying and you’re looking at managing those final days. | #19 |
| CMO3 | C: Cooperation and confidence | prerequisites for the successful implementation of end-of-life tools and frameworks in these settings. These include the cooperation of GPs with care homes [11] and the confidence of GPs in care home staff, | 28 |
| CMO3 | Context where mechanism may not fire – different attitudes | Difficulties arise between care home staff, GPs, multidisciplinary team members and families when there are competing accounts of who should lead care decisions at the end-of-life, misunderstandings, communication difficulties and different attitudes towards death and dying | 28 |
| CMO3 | M: Involving multidisciplinary team in QI (audit) | The results of the 05/6 audit, and the implications for patients, were also presented to the staff of all six care homes during a multidisciplinary study day to which GPs, District Nurses, OOH staff, care home managers and staff were invited | 29 |
| CMO3 | M: GP discusses ACP with resident, family, care home staff | One facet of anticipatory planning is discussion about preferred  place of care and death. This involves not only providing patients and families with the opportunity to express their wishes but also understanding the views of nursing and care staff. | 29 |
| CMO3 | O: More visits & more active role in ACP | The 10% rise in visits by GPs to nursing homes in 08/9 reﬂects a greater workload which on discussion with the partners is seen to reﬂect a more active role in anticipatory planning and end of life care. | 29 |
| CMO4 | C: Variability in care homes | Additionally homes vary in terms of resident type, size, management style and culture, which may all inﬂuence collaboration. | 30 |
| CMO4 | C: Variability in services | Practitioners working with nursing homes include general practitioners, district nurses and nursing and medical specialists in palliative care (SPC). However there is little research into collaboration between homes and these services …. | 30 |
| CMO4 | C: Variability in services | GP services remain disjointed and variable. (Home 29, survey) | 30 |
| CMO4 | C: GP with interest in frailty | if you do get a GP that has got an interest in, you know frailty, end of like care, you know holistic approach to medicine. Then I think, you know it’s a win, win if they are your care home lead | #21 |
| CMO4 | C: Collaboration | The focus is on organising and improving the quality of care for care home residents in the last year of life in collaboration with GPs, primary care and specialist palliative care teams. | 39 |
| CMO4 | C: Collaboration | our evaluation also revealed that improved collaboration was not a universal outcome, and it appeared that a minimum level of collaboration at baseline helped support GSFCH implementation, and retention in the programme. | 30 |
| CMO4 | C: GP delivering training (as part of interprofessional group) | Workshops are taught by trained facilitators from a variety of backgrounds in health and social care (e.g. GPs, palliative care specialists, social workers, nurses). | 31 |
| CMO4 | C: GP Leading national programme | The GSF was developed by generalist practitioners to address their own needs for support and education related to end-of-life care | 30 |
| CMO4 | C: GP leading national programme | Keri Thomas led the team which devised the Gold Standards Framework in care homes. | 30 |
| CMO4 | M: GP as leader | …leadership; galvanising change, being catalytic, is something that lots of people can do; it can be the visiting nurse, it can be your dietician who decides to do something. At least, going about improving all health and hygiene in a resident population. So lots of people can do the bright new things. I guess the task is to challenge them and say: can you sustain it and can you see this happening on a repeated basis over a long period of time… it doesn’t have to be general practitioners who do this, although they may be quite well positioned in some ways… | #11 |
| CMO4 | M: Facilitating trusted relationship | …the GSFCH programme helped to address a number of limiters to collaborative working, including some perceptions of unequal status and lack of trust between practitioners. This was achieved by providing nursing homes with frameworks for considering end-of-life care, relevant training, networking and support. | 30 |
| CMO4 | O: Interprofessional learning | They praised the expertise of the workshop facilitators (n=64), noted the benefits of interprofessional learning (n=36) | 31 |
| CMO4 | Context where mechanism does not fire – lack of support from GP | Concerns included GPs who respondents felt did not understand the GSFCH or palliative care, were slow to appreciate the beneﬁts of GSFCH, were not pro-active, who lacked trust in nurses, or who were reluctant to prescribe anticipatory medication. Comments from homes included: | 30 |
| CMO4 | Context where mechanism does not fire – lack of support from GP | Some managers indicated that while the majority of GPs were supportive of their efforts to improve end-of-life care, a minority were not. This presented practical problems, as nurses worked with GPs who had different approaches to end-of-life care. | 30 |
| CMO4 | Context where mechanism does not fire – lack of support from GP | Not all the practices that come into our home have been supportive of the GSF. (Home 55, survey)  GPs have been reluctant to participate.. and I have had received little co-operation. (Home 61, survey) | 30 |
| CMO4 | Context where mechanism does not fire – lack of support from GP | . . . we still don’t seem to have a brilliant communication with the actual GP that works with us. (Manager 84) | 30 |
| CMO4 | Context where mechanism may not fire – lack of support from GP | … you need a GP that’s cooperating. So the GP needs to take it as their responsibility. Because we needed to involve them - that was the difficulty… because of their time. .. | 39 |
| CMO4 | Context where mechanism may not fire – lack of action by care home | Problem of staff member who doesn't approve of providing high level of care and refuses to deliver ACP | #11 |

# Appendix references (for articles which have not been cited within the main text of the article)

1. Baqir W, Barrett S, Hughes J, Desai N, Riddle J, Laverty A. A clinico-ethical framework for multidisciplinary review of medication in nursing homes: a Health Foundation Shine project. 2013;21:64.

2. Baqir W. Improving medicine prescribing in care homes. In: Foundation TH, ed. *The Health Foundation.* Vol 2020. London: The Health Foundation; 2013.

3. Baqir W. Multidisciplinary review of medication in nursing homes: a clinico-ethical framework. In: Foundation TH, ed. *The Health Foundation*. London: The Health Foundation; 2018.

4. Baqir W, Barrett S, Desai N, Copeland R, Hughes J. A clinico-ethical framework for multidisciplinary review of medication in nursing homes. *BMJ Open Quality.* 2014;3(1).

5. Ballard C, Orrell M, Sun Y, et al. Impact of antipsychotic review and non-pharmacological intervention on health-related quality of life in people with dementia living in care homes: WHELD—a factorial cluster randomised controlled trial. *International Journal of Geriatric Psychiatry.* 2017;32(10):1094-1103.

6. Ballard C, Corbett A, Orrell M, et al. Impact of person-centred care training and person-centred activities on quality of life, agitation, and antipsychotic use in people with dementia living in nursing homes: A cluster-randomised controlled trial. *Plos Medicine.* 2018;15(2).

7. Badger F, Clifford C, Hewison A, Thomas K. An evaluation of the implementation of a programme to improve end-of-life care in nursing homes. *Palliat Med.* 2009;23(6):502-511.

8. Kinley J, Hockley J, Stone L, Dewey M, Hansford P, Stewart R. The provision of care for residents dying in UK nursing care homes. 2014;43:375-379.

9. Khunti K, Kinsella B. Effect of systematic review of medication by general practitioner on drug consumption among nursing-home residents. *Age Ageing.* 2000;29(5):451-453.

10. Hughes CM, Patterson S, Schweizer A. Information is care: the need for data to assess the quality of care in UK nursing and residential homes. *Expert Opin Drug Saf.* 2003;2(6):523-527.

11. Cox H, Puffer S, Morton V, Cooper C, Hodson J, Masud T. Educating nursing home staff on fracture prevention: a cluster randomised trial. 2008;37:167-172.

12. Patterson SM, Hughes CM, Cardwell C, Lapane KL, Murray AM, Crealey GE. A cluster randomized controlled trial of an adapted U.S. model of pharmaceutical care for nursing home residents in Northern Ireland (Fleetwood Northern Ireland study): a cost-effectiveness analysis. *J Am Geriatr Soc.* 2011;59(4):586-593.

13. Shah SM, Carey IM, Harris T, Dewilde S, Cook DG. Quality of chronic disease care for older people in care homes and the community in a primary care pay for performance system: retrospective study. *Bmj.* 2011;342:d912.

14. Madigan SM, Fleming P, Wright ME, Stevenson M, Macauley D. A cluster randomised controlled trial of a nutrition education intervention in the community. *J Hum Nutr Diet.* 2014;27 Suppl 2:12-20.

15. Iliffe S, Davies N, Manthorpe J, et al. Improving palliative care in selected settings in England using quality indicators: a realist evaluation. *BMC Palliat Care.* 2016;15:69.

16. Szczepura A, Wild D, Khan AJ, Owen DW, Palmer T, Muhammad T. Antipsychotic prescribing in care homes before and after launch of a national dementia strategy: an observational study in English institutions over a 4-year period. 2016;6:e009882.

17. Sadler E, Porat T, Marshall I, et al. Shaping innovations in long-term care for stroke survivors with multimorbidity through stakeholder engagement. *PLoS ONE.* 2017;12(5):e0177102.

18. Elvey R, Bailey S, Checkland K, et al. Implementing new care models: learning from the Greater Manchester demonstrator pilot experience. *BMC Fam Pract.* 2018;19(1):89.

19. Stewart K, Hargreaves C, Jasper R, Challis D, Tucker S, Wilberforce M. Provision and perceived quality of mental health services for older care home residents in England: a national survey. *International Journal of Geriatric Psychiatry.* 2018;33(2):364-370.

20. Stocker R, Bamford C, Brittain K, et al. Care home services at the vanguard: a qualitative study exploring stakeholder views on the development and evaluation of novel, integrated approaches to enhancing healthcare in care homes. *BMJ Open.* 2018;8(3):e017419.

21. Northumbria Healthcare NHS Foundation Trust, The Health Foundation. Shine – multidisciplinary review of medication in nursing homes. In: Specialist Pharmacy Service NHS, ed. *WHO Good Practice Repository.* Vol 20202018.

22. Northumbria Healthcare NHS Foundation Trust. *Shine 2012 final report. A clinico-ethical framework for multidisciplinary review of medication in nursing homes.* 2014.

23. Zermansky AG, Alldred DP, Petty DR, et al. Clinical medication review by a pharmacist of elderly people living in care homes--randomised controlled trial. *Age Ageing.* 2006;35(6):586-591.

24. Ballard C, Fossey J, Corbett A, et al. Impact of wheld intervention on neuropsychiatric symptoms, antipsychotic use and quality of life in people with dementia living in nursing homes: Acluster randomized trial. *Alzheimer's and Dementia.* 2016;13(7):P171.
